# Are Alternative Crops Needed for Future Sustainable Food Production?

**DOI:** 10.3390/plants15050804

**Published:** 2026-03-05

**Authors:** Jillian M. Lenné, David Wood

**Affiliations:** North Oldmoss Croft, Fyvie, Turriff AB53 8NA, UK; agrobiodiversity@gmail.com

**Keywords:** alternative crops, staple food crops, food and nutritional security, crop improvement

## Abstract

The momentum to reduce global reliance on a few staple food crops has been growing in recent years, partly fuelled by a belief that the situation is precarious and the world is at risk of a major food crisis. In this context, the perspective paper poses the overarching question: “Are alternative crops needed for future sustainable food production?” In responding to the question, it critically analyses the following: the risk of reliance on a few staple food crops; the importance of widely grown, preferred crops that are not considered global staple food crops; the confusion surrounding the term “alternative crops” and other crop categories; the positive and negative characteristics of some candidate alternative crops compared to staple and commonly grown crops; the dangers of promoting indigenous crops as alternative crops; the current and expanding knowledge base of the three most important staple food crops for stress tolerances and nutritional content to support future food production; and research needs for developing alternative crops in a declining funding scenario for agricultural research and development. The emphasis is on the Global South, where future needs are likely to be greater. It concludes that unless the existing staple and widely grown food crops face catastrophic limitations to future food and nutritional security under changing climates or other known risks, with current limited available funding for research, emphasis should be given to further improvement of staple and widely grown crops to cope with stresses. Alternative crops with useful additional traits could be available to complement staple crops rather than replacing them. Difficult choices may need to be made. Choices are likely to be country or regionally based, and cropping system-specific. It will be essential to consult with all actors—especially farmers—on the degree to which alternative crops have a role to play in contributing to future sustainable food production.

## 1. Context

Calls for the diversification of global cropping systems are anchored in concerns that humanity is precariously dependent on very few staple food crops—a highly risky scenario [1]. Three of these crops—rice, wheat and maize—provide around 50% of the total plant-derived energy consumed by humans globally. Wheat, rice and maize are staple foods for more than 5 billion people [2]. Of the current estimated global population of 8.3 billion, the number of hungry people is estimated at about 733 million—almost one in ten people [2,3]. An unexpected and significant reduction in the production of staple and commonly grown crops is likely to further increase the number of hungry people.

There is no doubt that changing climates will impact the food production potential of staple crops [4], although current scientific research is progressively mitigating these impacts through breeding and management, as discussed later in this paper. Additionally, recent conflicts in key crop production regions such as the Russia–Ukraine war [5,6] and increased tariffs on trade [7,8] are disrupting the production, supply of, and access to food. Escalating these already worrying developments into a potential full-scale global food crisis may scare countries into seeking immediate solutions, which may not be the best options in the long term.

Fowler et al. [9] define alternative as a mutually exclusive substitute or replacement. A common context of the growing call for “alternative crops” is to replace current staple food crops with “alternative crops”. The justification is that such crops are more resilient to changing climates and more nutritious than staple food crops and will therefore contribute to future sustainable food production [6,10,11]. Additional advantages highlighted for adopting alternative crops include reduced reliance on chemicals, enhanced soil health, increased tolerance/resistance to pests and diseases, and potential to diversify income through new market opportunities, among others [12]. In many cases, the claims are not supported by evidence, as scientific research needed to verify such claims has not been performed. Bearing in mind that food security is founded on both the **quantity of food** and its **nutritional content**, as also noted by Mavroeidis et al. (6)**,** two critical questions need to be addressed prior to the replacement of staple crops by alternative crops or any other crop options: first, whether the alternative candidate crop will produce as much or more food per area as the staple crop that it will replace, and second, whether a focus on nutritional traits may overlook naturally occurring anti-nutritional factors and even toxins, which reduce the absorption of minerals and protein and, in some cases, negatively affect health [13,14]. Most critical will be the development of rational and feasible strategies for the deployment of candidate alternative crops in appropriate farming systems supported by farmers, markets and consumer preferences, especially those aimed at improving fairness and enhancing smallholder livelihoods [15].

The difficulties in achieving such objectives should not be underestimated [6,16], especially with funding support for agricultural research for development in decline, as discussed below. It should be noted at the outset that this paper is not about the diversification of cropping systems *per se.* Several papers already published in *Plants* have comprehensively addressed this issue [17,18]. This paper is specifically focused on the growing call for the replacement of staple crops with alternative crops. This may lead to diversification in cropping systems depending on the circumstances.

## 2. Objectives

This perspective paper analyses the rationale and feasibility of growing calls for alternative crops to replace staple food crops for future food production. It considers the need to address threats to future food production and security with rational options rather than arguably questionable and knee-jerk solutions.

We first consider the level of risk of reliance on a few staple food crops. Second, we highlight widely grown, preferred and consumed crops, mostly from the Global South, which are not considered to be global staple food crops even though they are often regional or national staples, and examine their role in reducing reliance on staple crops and contributing to sustainable crop production. Third, we explore what is meant by “alternative” crops and the confusion surrounding the interpretation of the term in line with the growing number of different, overlapping and ambiguous crop categories. Fourth, we analyse the characteristics—both positive and negative—of some of the proposed alternative crops in comparison to commonly grown crops in the same crop groups, with emphasis on cereals and legumes. Fifth, we emphasize the dangers of promoting indigenous crops as alternative crops due to the usually negative impact of indigenous pests and diseases. Sixth, we summarize the current knowledge base of the three most important staple food crops for stress tolerances and nutritional content in the context of providing future solutions to food production. Seventh, we briefly look at research needs and funding. Finally, we suggest that a more feasible and scientifically sound approach would be to view useful alternative crops as complementary crops to staple and/or widely grown and preferred food crops, selected primarily to add additional advantages—not just diversity—to the cropping system for sustainability and, importantly, future food production.

## 3. How Risky Is the Apparent Reliance on a Small Number of Staple Food Crops?

Seventy years ago, the post-apocalyptic science fiction novel *The Death of Grass* was published [19]. The plot concerns a virus that kills all *Gramineae*, including the staple cereal crops wheat, rice and maize, threatening famine. The world rapidly descends into anarchy. Unfortunately, some recent publications on the reliance on a small number of staple food crops elevate the apparent risk towards a similar “precarious risk to humanity” [1,7]. It is therefore crucial to rationally assess the current level of risk. The major staple food crops wheat and rice were domesticated around 8–9000 BC, with maize being domesticated slightly later at around 7000 BC [20,21]. It is estimated that these crops became global staples around the Bronze Age. Over the past 5000 years, these three crops have been subjected to multiple climate changes, pest and disease outbreaks (some are recorded in the Bible [21]) and geopolitical events, including two recent world wars. They have remained staple food crops due to their resilience, which in the past 100 years has been increasingly supported by the global scientific community conducting research on heat, drought and salinity tolerances for over 50 years, as well as by research over the past 30 years on improving the nutritional quality—protein, minerals and vitamins—generating many improved varieties. Not only are these crops resilient, but they have also benefited from significant risk mitigation. Furthermore, integrating crops with complementary traits into existing systems further reduces risks. Most importantly, major staple crops are already complemented by many other widely grown and consumed crops in all crop groups, namely cereals, roots and tubers, legumes, oil seeds, vegetables, fruits and nuts. Over-exaggerated risks and scare-mongering need to be placed in perspective based on existing approaches to address threats to future food production. It is not possible to ensure future food production against unknown threats.

## 4. Widely Grown Non-Staple Food Crops Reduce Risks, Diversify Food Systems and Reduce Reliance on Staple Food Crops

Widely grown non-staple food crops, both nationally and regionally, include pearl and finger millets, sorghum, many root and tuber crops, legumes such as cowpea, common bean, pigeon pea, groundnut, and many vegetables and fruits [22]. Most cropping systems in the Global South rely on these food crops, with the main exception being much of Asia, which predominantly relies on rice. Root and tuber crops are important food crops for over 1 billion people in the Global South, representing 5.3% of global consumption of all crops, including cassava (2.6%), potato (1.7%), sweet potato (0.6%), and yam (2.4%) [22]. They account for roughly 40% of the food eaten by half the population of SS Africa. As well as providing carbohydrates for energy, they are high in calcium and vitamins (C and A). Cassava is a staple food for around 500 million people [22]. In much of Africa, root and tuber crops would be considered staple food crops. These commonly grown food crops provide an important option to reduce risks from reliance on staple cropping systems and are already making a sizeable contribution to sustainable crop production.

## 5. What Are Alternative Crops?

Sumberg and Giller [23] emphasized in their paper on “What is conventional agriculture?” that adjectives deserve serious consideration, especially with regard to how they are used in both the scientific and popular literature. This is also the case for crop categories that are often poorly defined, ambiguous and confusing. These include staple, widely grown, indigenous, neglected, underutilized, traditional, alternative, introduced, service, companion, etc. Box 1 attempts to provide some clarity, especially for alternative crops, the subject of this paper. Many crops have been described by more than one of these terms depending on the circumstances in which they are grown in agroecosystems.

Box 1 **Crop categories (Crop category descriptions compiled by the authors from a range of sources, including from many citations in the paper. There are no internationally defined definitions of these categories) used in both scientific and popular literature.**

Staple—crops consumed daily, forming the dominant part of global dietary energy and nutrients, e.g., rice, wheat, and maize

.

**Widely grown**—crops consumed daily forming a dominant part of national or regional dietary energy and nutrient needs, e.g., cassava (SS Africa and Latin America), pearl millet and sorghum (SS Africa)

.

**Indigenous**—crops native to a specific country or region, often grown by smallholder farmers for household consumption

.

**Neglected**—crops native to a specific country or region that have largely been ignored by agricultural research

.

**Under-utilized**—also called orphan or minor crops—generally local crops cultivated for household consumption with potential for development

.

*Note: neglected and under-utilized crops are often considered the same and known by the acronym NUS*

.

**Traditional**—often classed in the same group as NUS crops—indigenous or locally grown crops cultivated by smallholders for household consumption for a long period—sometimes also called heirloom crops

.

**Alternative**—*not internationally defined*—crops used to replace staple crops, e.g., quinoa, amaranth, emmer, einkorn and spelt for staple cereals

.

**Introduced**—crops introduced from one region to another, commonly inter-continentally, e.g., cassava from South America to SS Africa, potato from South America to Europe

.

**Service**—crops grown to provide ecological, environmental and/or soil-enhancing benefits in agroecosystems—may or may not be food crops

.

**Companion**—crops often intercropped with staple and/or widely grown crops to achieve mutual benefits such as improved pest management and/or soil conditions, e.g., cereal-legume intercrops

Although there have been past and more recent calls to give greater consideration to underutilized, neglected, traditional and/or indigenous crops in the Global South [16,24], the focus now appears to have shifted to calls for alternative crops. As a result, there is considerable confusion about what alternative crops are as there is no strict classification of a group of crops as **alternative** [6], and many publications cited in this paper have considered the term to be the same as underutilized, neglected, traditional and/or indigenous. The simplest definition of an alternative crop is any crop that is not currently grown in the target cropping system [25]. As this could include underutilized, neglected, traditional and/or indigenous crops, as well as improved varieties of reintroduced staple food crops, it is not especially helpful in understanding what an alternative crop is. Other definitions include: crops (re)introduced in a particular geographic area due to their potential high value or other benefits to the farming systems of that area [26]; crops that can be introduced into a new cropping system to replace traditional crops that are usually more susceptible to biotic and abiotic stress [27]; opportunity crops with great unrealized potential to improve food and nutrition security in the context of climate change with a focus on Africa [10,28]; and nutritional crops that are under-researched in Africa (a mix of indigenous and exotic annuals and perennials) [29,30].

In some cases, further confusion is created by the inclusion of widely grown and often well-researched crops as alternative and/or underutilized [10,30]. Some of these crops are grown over millions of hectares in various regions of the world, although not always in cropping systems dominated by staple food crops. In such regions, they are considered to be staple food crops, e.g., sorghum and millets in Africa and South Asia and cassava and yam in Africa. It would be much less confusing if all of the different categories of these crops were called “food crops” which could then be qualified by descriptions of their traits.

## 6. Selected Candidate Alternative Crops: Positive and Negative Characteristics

Although it is outside the scope of this paper to analyse all the crops being proposed as alternative crops, the paper considers a selection of crops that have been identified by a number of authors as candidate alternative crops in recent years and are supported by the literature cited in Table 1. These include cereals, pseudo-cereals, and legumes. Most have been proposed as alternative crops based mainly on nutritional content and resilience to changing climates (principally heat and/or drought tolerance). Important factors such as yield potential and the existence of anti-nutritional factors have rarely been considered in their promotion. Table 1 lists some of these crops together with their positive and negative characteristics. The importance of crop x biotic problems (pests and diseases) in different countries could not be included due to the limited information in the literature. However, one aspect of alternative indigenous crops, pests and diseases is discussed in a separate section below.

### 6.1. Nutritional Content

All twelve crops in the three crop groups are nutrient-rich in minerals and fibre, with most also being noted for higher and/or more complete protein content than staple cereals (Table 1), although direct comparisons are rarely made. In addition, five of the seven selected cereals and pseudo-cereals are gluten-free, which is of value in specialist diets for those with gluten intolerance. However, at the same time, eleven of the twelve crops also possess anti-nutritional factors (Table 1), which reduce their nutritional value. These include phytates, saponins, tannins, alkaloids, protease and trypsin inhibitors, which disrupt digestion and reduce absorption of protein and minerals. Seed processing, such as soaking, sprouting, cooking and fermentation (e.g., fermented injera pancakes from teff) can reduce the concentrations of anti-nutritional factors. Pseudo-cereals are unlikely to replace staple cereals due to the presence of compounds that confer undesirable organoleptic and technological characteristics to their products [42].

Two legumes are noted for toxins: lablab contains cyanogenic glycosides, while grass pea contains β-N-oxalyl-l-α,β-diaminopropionic acid (β-ODAP) [54,64]. Cyanogenic glycosides are concentrated in mature dry lablab seed and cause cyanide poisoning—vomiting, convulsions and potentially death. β-ODAP, found in grass pea seed, causes the neurodegenerative disease lathyrism—irreversible paralysis. Furthermore, quinolizidine alkaloids, especially cytisin and anagyrin, cause poisoning in livestock [65]. Seed processing, including soaking, prolonged boiling with several changes in water, and fermentation can lower toxin levels.

The additional processing of alternative crops to remove anti-nutritional factors and toxins is a significant burden on smallholder farming households in the Global South [66]. It is labour- and time-intensive, suffers energy constraints, and can reduce food quality through loss of water-soluble minerals and vitamins. Furthermore, many smallholders may lack the awareness and/or knowledge of the presence of anti-nutritional factors in crops newly introduced into their cropping systems. Because they do not possess high levels of anti-nutritional factors or toxins, most staple and widely grown cereals and legumes do not inflict the same burden of processing on smallholders. In comparison, past and current research efforts have reduced anti-nutritional factors in staple and widely grown crops (for example: [67,68,69,70]).

### 6.2. Resilience to Changing Climates—Drought and Heat Tolerance

As the majority of candidate alternative crops in Table 1 evolved in countries/regions with low rainfall and poor soils, not unexpectedly, they have noted drought and poor soil tolerance (Table 1). Some, such as lablab, also have heat tolerance, while grass pea has salinity tolerance. Again, comparisons with staple and widely grown food crops are rarely made, although there is strong evidence to support their own resilience to climate change. For example, cereals, including pearl and finger millets, widely grown by smallholders in drier areas of the Global South, are drought tolerant [71], while legumes, such as widely grown cowpea and pigeon pea, are noted for drought tolerance [72,73]. Similarly, sorghum and pearl millet have high levels of heat tolerance [74,75]. Cowpea is also heat tolerant but vulnerable at the flowering stage, which is being addressed through innovative breeding approaches [76]. Similarly, recent scientific advances in pigeon pea through speed breeding have greatly increased its heat tolerance up to 45 °C [77].

Widely grown and smallholder-preferred crops in the Global South are already conferring drought and heat tolerance without introducing alternative crops with negative anti-nutritional traits. Although confronting such stresses in crops is complex, the scientific understanding of strengthening the tolerance of major crops is substantial [78] in contrast to the current lack of knowledge for alternative crops. There is no guarantee that candidate alternative crops, currently resilient to prevailing climates, will continue to be resilient in the coming years. There is therefore a critical need for more research to understand the potential of candidate alternative crops to tolerate greater heat and other stresses as climatic changes are expected to continue to grow.

### 6.3. Poor Soil Tolerance and Yield

Tolerance of poor soils is often promoted as a positive characteristic of alternative crops, but invariably, this is manifest in low yields—less than 1 t/ha—as illustrated in Table 1. However, low yields in some alternative crops can also be caused by a lack of scientific input by crop breeders to a) select the best genotypes among the diversity present and b) develop improved higher-yielding varieties that do not suffer from shattering and lodging just as has already been done in staple food crops. The domestication of wheat eight thousand years ago rescued it from its shattering wild progenitor [20], while the development of semi-dwarf rice and wheat in the 1960s removed the problem of lodging leading to the Green Revolution [79]—the semi-dwarfing character is now being developed in maize [80]. The ability of alternative crops to grow in poor soils should not be considered a benefit if the result is low yields in food production. Dependence on low-yielding alternative crops will inevitably result in bringing more land into cultivation to grow enough food to achieve food security, with associated risks to wild biodiversity [81].

Recently, interest has revived in heritage wheats—emmer, einkorn and spelt—as potential alternative crops (Table 1). Although still grown in parts of North Africa, in Europe and other countries, they were mainly grown for the boutique health food market [37,82]. These wheats have drought tolerance and can grow in poor soils, but in most years, yields are about a third of those of bread wheat. For perennial wheat such as kernza, there is a basic trade-off between the carbon and nitrogen needs of the perennial plant and high grain yields; achieving one negates the other, as grain breeders have long found [39]. The first commercial perennial grain, kernza, yields a third as much as bread wheat, with declining yield after the first year [40]. Finally, candidate alternative legume crops (Table 1) are all lower yielding than commonly grown legumes such as cowpea, common bean, pigeon pea and groundnut, which have been subjected to sound scientific research over many decades for crop improvements [83].

When confronted with a choice between a staple and/or widely grown and preferred food crop and a relatively unknown alternative crop with anti-nutritional factors and low yields, it will be difficult for smallholders in the Global South to adopt such crops, especially if they are unaware of their nutritional value and concerned about market potential, as noted by Aryal and Lopez-Lavelle [84].

## 7. Risks of Promoting Indigenous Crops in Africa as Alternative Crops

The need to produce more food in Africa has stimulated interest in the promotion of a number of species indigenous to Africa as alternative crops. Some of these—sorghum, pearl and finger millets, cowpea, pigeon pea, okra and white yam—are widely grown, important, and much-researched food crops, including the development of improved varieties with resistance to local pests and diseases. Other African crops such as fonio millet, Kersting’s groundnut, Livingstone potato, and many leafy vegetables and fruit-producing tree crops, which have largely been neglected by both smallholders and scientists, are being promoted as alternative crops. Part of the interest in indigenous crops in Africa is an arguably misplaced belief that introduced crops such as maize, common bean, cowpea, cassava, sweet potato and others were imposed on smallholder farmers by colonialism [85]. As a result, some activists believe there is a need to decolonize agriculture by rejecting the legacies of colonial systems and reclaiming cultural identity and local knowledge, which manifests as the need to promote African crops [86,87]. This ignores the probability that smallholder farmers made sensible decisions to adopt introduced crops as they gave higher yields, were less affected by local pests and diseases, and were preferred by consumers.

The Vision for Adapted Crops and Soils (VACS) carried out a comprehensive modelling exercise based on adaptation indicators, with a strong focus on nutrition and climate resilience to identify candidate crops [10]. With emphasis on Africa, sixty crops were selected among all crop groups, with over thirty being indigenous. Similarly, the African Orphan Crops Consortium selected 100 nutritious crops on the basis of being under-researched in Africa [30], over fifty of which are indigenous, including a large number of fruit-bearing trees newly classed as “crops”. Table 1 acknowledges this interest by including six crops which are indigenous to Africa: teff, fonio millet, amaranth (some species), lablab, Bambara groundnut, and African yam bean.

Indigenous crops are generally well-adapted, often considered “locally adapted”, to local abiotic conditions (seasons, day length, soils, temperature and water availability), but they are locally constrained by their indigenous pests and diseases and are therefore vulnerable to yield and quality losses. This topic has already been critically analysed in several previous papers [18,88,89]. Notwithstanding the significant additional challenges of utilizing indigenous crops for Africa’s future food security including lack of research and development, poor market potential, inadequate policy frameworks, insufficient extension services, and lack of awareness compounded by sociocultural choices for staple crops like maize [90], improving these recently promoted indigenous crops for tolerance/resistance to local *biotic* constraints would be a monumental task [88] and probably take many years. The *relative* freedom due to the escape of introduced crops from the constraints of local pests and diseases is a well-established fact [18,89]. This is a major reason why most countries in Africa depend on introduced crops for food security, with as much as 85–90% of the value of production [89]. Wide promotion of indigenous crops in Africa as candidate alternative crops is a significant risk to food production and security.

The outstanding success of introduced crops for food production and security in Africa could be expanded to diversify and nutritionally enhance their cropping systems without concerns over pest and disease problems. South America has a diversity of nutritious crops that have not yet been introduced to Africa. For example, Andean roots and tubers including oca (*Oxalis tuberosa* Molina), ulluco (*Ullucus tuberosus* Caldas), mashua (*Tropealum tuberosum* Ruiz & Pav.), yacon (*Smallanthus sonchifolius* (Poepp.) H. Rob.), maca (*Lepidium meyenii* Walp.), and arracacha (*Arracacia xanthorrhiza* Bancroft) provide energy, fibre and minerals and grow in marginal conditions [91,92]. The success of potato in Africa to date supports this option. Similarly, although some tropical fruit crops have already been successfully introduced to Africa, such as avocado, passion fruit, tree tomato, tree grape, acai and guava, South America boasts a huge diversity of delicious fruit crops with potential to contribute to food and nutritional security in Africa such as peach palm (*Bactris gasipaes* Kunth.), lucuma (*Pouteria lucuma* (Ruiz & Pav.) Kuntze), lulo (*Solanum quitoense* Lam.), cupuacu (*Theobroma grandiflorum* (Willd. Ex Spreng.) K Schum.), and soursop (*Annona muricata* L.), among others [93].

## 8. Staple Food Crops: Stress Tolerance and Enhanced Nutritional Content

Table 2 summarizes selected recent literature on stress tolerance and nutritional enhancement research in the staple crops wheat, rice and maize. The improvement of these staple food crops, as well as of other widely grown food crops for tolerance to heat, drought and salinity, has been in progress for over fifty years with considerable success, long before the current concerns about resilience to climate change [94,95]. Similarly, over the past thirty years, research on improving the nutritional quality—protein, minerals and vitamins—in staple food crops has generated many improved varieties [96,97,98]. Initially, conventional plant breeding was implemented, but the focus has now shifted to using genomic analysis and biotechnology coupled with high-throughput phenotyping to more efficiently target key genes and speed up the process of developing superior stress-tolerant and nutritionally enhanced varieties [99,100,101,102]. A comprehensive knowledge base and a corpus of skilled researchers support this continued improvement in staple and widely grown crops for food production, a luxury not yet available to alternative crops. Much of this is through public sector breeding and not through the private sector. Many improved varieties are already grown by smallholder farmers [97,98,103]. For example, to date, 37 biofortified Fe, Zn, protein and provitamin A-rich rice varieties are under commercial cultivation (16 in India and 21 in the rest of the world).

Furthermore, research is also well-advanced to ameliorate the negative effects of heat and drought stress in staple food crops by agronomic management to complement improvement efforts [4,113,114]. Adjustments in crop selection, soil management, and fertilizer practices have shown promise in sustaining crop yields under heat stress. The appropriate management choice, however, requires a deeper exploration of the underlying mechanisms behind the observed yield reductions. Combining crop improvement and innovative management methods supports the present and future ability of staple and widely grown crops to contribute to future sustainable food production. The topic merits an in-depth analysis complementary to the focus of this paper on breeding.

## 9. Research Needs and Declining Funding

### 9.1. Research Needs for Utilizing Alternative Crops for Future Food Production

In contrast to staple and widely grown food crops, many candidate alternative crops lack the necessary research base for productive utilization. Chivenge et al. [16] highlighted the lack of knowledge on agronomy, crop improvement, post-harvest handling and value addition, as well as the lack of linkages of farmers to markets. Mavroeidis et al. [6] noted the need for region-specific studies on environmental and pedoclimatic niches, understanding crop diversity and implementing breeding programmes, understanding of regional food preferences and societal benefits, as well as the need for raising awareness. Aryal and Lopez-Lavelle [84] stressed the limited technical knowledge, availability of planting material, inadequate post-harvest processing and under-developed marketing mechanisms to be key barriers to adoption of quinoa in North Africa. Mmbando et al. [90] emphasized the lack of research and development for crop improvement, lacklustre markets, inadequate policy frameworks, lack of awareness and the difficulties in changing sociocultural preferences for preferred crops such as maize and rice. Finally, Pasipanodya et al. [58] raised the need for a dedicated crop improvement programme for Bambara groundnut in Namibia, which is likely to be the case for other alternative crops that have not yet benefited from crop improvements. In spite of the beneficial traits of some candidate alternative crops, collectively, the research and development required for their effective utilization as crops that will contribute to food and nutritional security is substantial.

### 9.2. Current Funding Scenario for Agricultural Research for Development

The funding for international agricultural research has been significantly declining since 2024 (from 9% in 2024 to 17% in 2025), primarily driven by major cuts in Overseas Development Assistance (ODA) from key donor nations, particularly the US, UK, Germany, and France [115,116]. The Russia–Ukraine war has exacerbated these cuts further as governments divert funds to bolster defence spending [117,118]. The most recent and significant decline was the dismantling of USAID in early 2025. This resulted in the termination of numerous research projects between US universities and their international and national partners in the Global South, affecting crop research especially for climate adaptation and food production in Africa, Asia and Latin America [119,120,121]. Additionally, it had a serious impact on CGIAR’s research (formerly the Consultative Group for International Agricultural Research), the largest and most successful network of agricultural research centres working in the Global South [122]. Of relevance, funding cuts have placed ongoing crop improvement of even the major staple food crops—wheat, rice and maize—under significant pressure [123]. Most importantly, the long-term foundational research funding necessary for crop research is becoming more difficult to sustain.

The immediate consequences are closure of projects, damaged partnerships, loss of experienced scientists responsible for developing new innovations for addressing the challenges of climate change, threats from pests and diseases, and the development of more nutritious and productive crop varieties. The longer-term consequences are a slowing in the development of new technologies for productivity growth and disruption to efforts to reduce food insecurity in the Global South. It is also likely that donor countries will be impacted by the loss of research expertise, which also benefits their own agricultural production.

In such a scenario, there is unlikely to be additional funding for the monumental research effort needed to improve alternative crops unless this is diverted from the shrinking pool of funding for staple food crops. Difficult choices will be necessary.

## 10. Concluding Remarks

This perspective paper posed the over-arching question: “Are alternative crops needed for future sustainable food production?” It uncovered two underlying questions that needed to be answered in order the give an informed response to the over-arching question. First, will the candidate alternative crops replace or be complementary to the existing crops? Second, do the candidate alternative crops offer additional beneficial traits for future sustainable food production? Answers to these underlying questions will be critical to developing appropriate strategies for deploying alternative crops in existing cropping systems.

In the case of the first question, unless the existing staple and widely grown crops face serious risks for future food and nutritional security under changing climates, emphasis should be given to (a) further improve them based on sound science to cope with stress and (b) complement them with alternative crops rather than replacement. Polarity based on replacement inevitably leads to paralysis with stagnating decision making. When factions are so constrained by their vision of the best solution to potential food crises, it becomes impossible to see other points of view. Solutions should not rely on diversity for its sake but on diversity for its function. The deployment of alternative crops is likely to be complementary in existing cropping systems, offering additional benefits to future sustainable food production.

With regard to the second question, the paper has demonstrated in detail (Table 1) that candidate alternative crops have positive and negative traits. They are usually nutritionally rich but also possess anti-nutritional factors that reduce their nutritional value and require resource- and time-consuming processing. They are often able to grow in marginal conditions and have tolerances to abiotic stresses, but this is manifest in low yields. Furthermore, the knowledge base to support their effective utilization for food production and security is often in its infancy. In contrast, the century of research support for staple and widely grown crops has founded a productive and increasingly resilient global food supply for most of humanity.

Unfortunately, the current declining funding and future uncertainties for agricultural research and development mean that we face difficult choices. The right choices will be paramount to answering the over-arching question. Crop choices may well be country or regionally based and cropping system-specific. Such choices—staple and/or alternative—should primarily be taken by farmers based on their knowledge of the suitability to the prevailing environment and benefits to their families including food, nutrition, marketability and profitability. Furthermore, their attractiveness can be influenced by research and development as well as policy support. At the same time, it will be essential to consult with all actors—farmers, consumers, and governments—about the degree to which alternative crops have a role to play in contributing to future sustainable food production.

## Figures and Tables

**Table 1:** Selected alternative crops: positive and negative characteristics.

Crop	Positive	Negative	Sources
**Cereals:**			
Teff (*Eragrostis tef* (Zucc.) Trotter): mainly grown in Ethiopia and Eritrea and also as a forage in USA, Australia and South Africa; considered a health food in the Global North	Nutritious (rich amino acid and mineral profile), gluten-free, drought and poor soil tolerant, dual purpose	Low yields due to small seed size, shattering, lodging, yields 0.7–2.5 t/ha (depending on inputs), anti-nutritional phytate, tannins, protease inhibitors limit protein and mineral absorption	[31,32,33]
Fonio millet (*Digitaria exilis* (Kippist) Stapf.): mainly grown in West Africa as a smallholder crop	Nutritious (minerals, fibre), Gluten-free, drought and poor soil tolerant	Very low yields due to small seed size, shattering, lodging, low yields 0.2–2 t/ha (depending on inputs), anti-thyroid flavonoids, 25% losses in post-harvest processing	[34,35]
Emmer (*Triticum turgidum* ssp. *dicoccum* (Schrank ex Schubl.) Thell.), Einkorn (*Triticum monococcum* L.) and Spelt (*Triticum spelt* L.): heritage wheats mainly grown in north Africa and Europe	Nutritious (protein, minerals, fibre), drought and poor soil tolerant, dual purpose	Medium yields 3–5 t/ha comparable to wheat in poor years (wheat 7–9 t/ha), phytate, tannins, polyphenols reduce bioavailability of minerals	[36,37]
Kernza (perennial wheat) (*Thinopyrum intermedium* (Host) Barkworth & D R Dewey): developed by the Land Institute, USA; currently not widely cultivated	Nutritious (protein, minerals, fibre), drought tolerant, dual purpose	Low yields 0.1–1.5 t/ha, inconsistent harvests	[38,39,40,41]
**Pseudo-cereals:**			
Quinoa (*Chenopodium quinoa* Willd.): an Andean staple food crop now grown in over 100 countries for its nutritive value	Nutritious (protein, minerals, fibre), gluten-free, drought, salinity and poor soils tolerant	Shattering under heat stress, low to medium yields 1–3 t/ha (depending on inputs), antinutritional factors phytates and saponins, post-harvest processing complex	[42,43,44,45,46]
Buckwheat (*Fagopyrum esculentum* Moench): native to SW China and Himalayas now widely grown in Europe, Americas and Russia	Nutritious (protein, minerals, fibre, flavonoids), gluten-free, poor soils tolerant	Shattering, lodging, sensitive to heat at seed set, uneven ripening, low yields 1–2.5 t/ha (depending on inputs), labour-intensive harvesting and processing, antinutritional factors (tannins, phytates, protease inhibitors) disrupt digestion	[42,47,48]
Amaranth (*Amaranthus* spp.): widely distributed in warm temperate to tropical areas	Nutritious (protein, minerals, fibre), gluten-free, drought tolerant, dual-purpose (both grain and leaves)	Shattering, low to medium yields 1–4 t/ha (depending on inputs), antinutritional factors (tannins, saponins, phytates) disrupt digestion	[42,49,50,51]
**Legumes:**			
Lablab/Hyacinth bean (*Lablab purpureus* (L.) Sweet): global cultivation as a food, forage and cover crop	Nutritious (protein, minerals, fibre), drought and heat tolerant, multiple-purpose, N-fixing	Low to medium yields 0.6–2.5 t/ha, with forage yields 1–2 tDM/ha, antinutritional/toxic cyanogenic glycosides	[52,53,54]
Bambara groundnut (*Vigna subterranean* (L.) Verdc.): West African crop spreading to East and Southern Africa	Nutritious (protein, minerals, fibre), drought and poor soil tolerant, N-fixing	Low yields 0.6–1 t/ha (depending on inputs), antinutritional factors (tannins, phytates, trypsin inhibitors) limits protein and mineral absorption	[16,55,56,57,58]
African yam bean (*Sphenostylis stenocarpa* (Hochst. Ex A Rich.) Harms.): native to tropical Africa and widely cultivated in West and Central Africa	Nutritious (protein, minerals, fibre), dual harvest (beans and tubers), N-fixing, grows in sub-humid to humid conditions	Low yields 0.3–2 t/ha (beans), 1–2 t/ha (tubers), not drought tolerant, antinutritional factors (saponins, tannins, oxalate, phytates, glycosides) limits protein and mineral absorption, unacceptable taste, prolonged cooking time, demanding management requirements	[59,60]
Tarwi (*Lupinus mutabilis* Sweet.): an Andean native being promoted globally as a protein-rich food	Nutritious (protein and seed oil), dual purpose, drought and poor soil tolerant, grows at high altitudes, N-fixing	Low to medium yields 0.4–1.5 t/ha (depending on inputs), heat-stable toxins (bitter quinolizidine alkaloids), seed must be soaked for several days before cooking and eating	[61]
Grass pea (*Lathyrus sativus* L.): mainly cultivated in South Asia as a food and feed crop	Nutritious (protein, minerals, fibre), stress-tolerant (drought, cold, salinity), N-fixing	Medium yields 1–2 t/ha, toxic due to neuroexcitatory β-N-oxalyl-l-α,β-diaminopropionic acid (β-ODAP), the cause of neurodegenerative disease—lathyrism—seeds must undergo prolonged soaking and boiling to reduce toxins	[62,63,64]

**Table 2:** Development of staple crop varieties with tolerances to stress and enhanced nutritional content.

Character	Crop	Sources
**Stress Tolerance**		
Heat	Wheat, rice, maize	[78,94,95,99,101,104,105,106,107]
Drought	Wheat, rice, maize	[94,95,104,105,106]
Other: Salinity, Flooding, Cold	Rice, maize	[105,108]
**Enhanced nutritional content**		
Minerals—Fe, Zn	Rice, wheat, maize	[97,103,108,109,110,111,112]
Vitamins—β-carotene	Rice	[103,110]
Protein—Lysine, Tryptophan—Quality Protein Maize	Maize (contains 19% more iron, 39% more zinc, and 30–80% more lysine and tryptophan than traditional maize)	[96,97,100]

## Data Availability

No new data were created or analyzed in this study.
